# 2688. Characteristics of joint infections in solid organ transplant recipients

**DOI:** 10.1093/ofid/ofad500.2299

**Published:** 2023-11-27

**Authors:** Victor P Abdow, John H Fraker, Rebecca Kumar, Ronald M Beaulieu

**Affiliations:** Georgetown University School of Medicine, Washington, District of Columbia; MedStar Georgetown University Hospital, Washington, District of Columbia; Georgetown University Medical Center, Washington, District of Columbia; MedStar Georgetown University Hospital, Washington, District of Columbia

## Abstract

**Background:**

There are sparse data surrounding joint infections in solid organ transplant (SOT) recipients, and this population is not specifically addressed in the Infectious Diseases Society of America (IDSA) Clinical Practice Guidelines. We aim to provide microbiologic and treatment data to help inform future guidelines on managing joint infections in this unique patient population.

**Methods:**

We retrospectively reviewed all adult SOT recipient admissions in our ten-hospital system for native or prosthetic joint infection between January 2015 and December 2021. Eighty-one patients were included based on ICD-9 or ICD-10 codes and were excluded if they were < 18 years old, did not have a SOT on immunosuppression, were transferred prior to treatment, or did not have a large joint infection (total n=33).

**Results:**

There were 33 joint infections in 25 patients. The overall median time from transplant to joint infection was 3.7 years; heart and multivisceral occurred earlier (1.3 and 0.8 years, respectively). More than 77% of native joint infections required surgical intervention (n=17); 16 required washout. All 11 prosthetic joint infections required surgical intervention: two-stage revision was required in 7 of these cases, and debridement, antibiotics and implant retention (DAIR) procedure was performed only once. Just 15% of patients (n=5) had a resistant organism; the most common was methicillin-resistant Staphylococcus aureus (n=3). The median duration of antibiotics was 6.0 weeks. Only 42% of patients had resolution of the joint infection (n=14). Other outcomes included chronic suppression (n=7), recurrence (n=5), new infection (n=3), death (n=3), and amputation (n=1).

**Figure:**
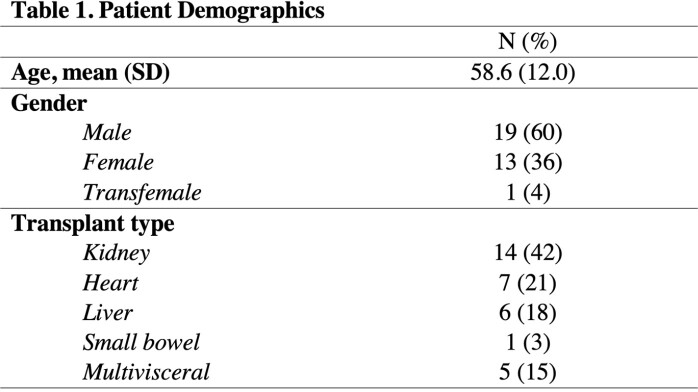

**Figure:**
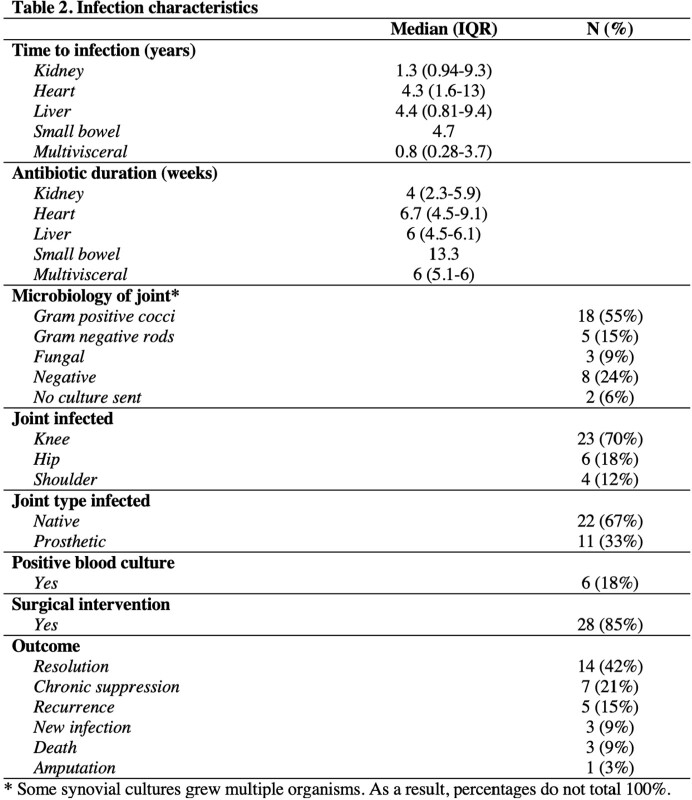

**Figure:**
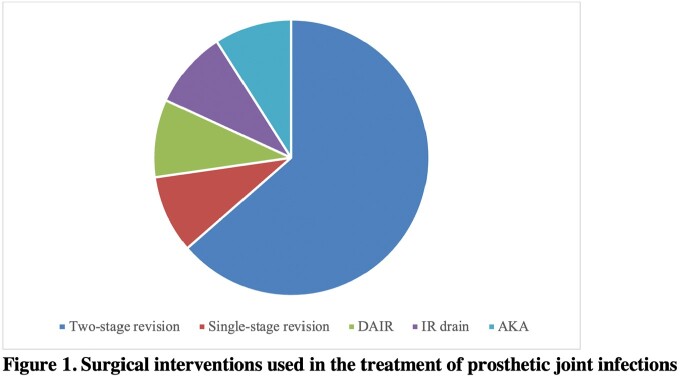

**Conclusion:**

This study found that joint infections typically occur more than a year out from transplant. IDSA guidelines for surgical management of prosthetic joint infections advocates for DAIR or single-stage revision where feasible. However, most of our SOT patients required two-stage revision, suggesting a possible need for more aggressive intervention in this population. Additionally, most patients did not have multi-drug resistant organisms, indicating that multi-drug resistant coverage (i.e. carbapenems) is not necessarily needed in this population.

**Disclosures:**

**Rebecca Kumar, MD**, Astra Zeneca: Grant/Research Support|Astra Zeneca: Honoraria|Regeneron: Grant/Research Support

